# A case of resected anaplastic carcinoma of the pancreas producing granulocyte-colony stimulating factor with literature review

**DOI:** 10.1186/s40792-024-02008-3

**Published:** 2024-09-05

**Authors:** Norio Kubo, Shigemasa Suzuki, Takahiro Seki, Shunsaku Furuke, Naoki Yagi, Takashi Ooki, Ryusuke Aihara, Akira Mogi, Yuka Yoshida, Kenji Kashiwabara, Yasuo Hosouchi, Ken Shirabe

**Affiliations:** 1 Department of Surgery, Gunma Prefecture Saiseikai Maebashi Hospital, 564-1 Kamishinden, Maebashi, Gunma 371-0821 Japan; 2 Department of Pathology, Gunma Saiseikai Maebashi Hospital, Maebashi, Japan; 3 Division of Hepatobiliary and Pancreatic Surgery, Department of General Surgical Science, Graduate School of Medicine, Gunma University, Maebashi, Japan

**Keywords:** Anaplastic carcinoma, Granulocyte-colony stimulating factor, Undifferentiated carcinoma, Pleomorphic-type

## Abstract

**Background:**

Granulocyte colony-stimulating factor (G-CSF)-producing tumors have been reported in various organs, and the prognosis of patients with G-CSF-producing pancreatic cancers is particularly dismal. In this report, we present a case of G-CSF-producing anaplastic carcinoma of the pancreas (ACP), characterized by early postoperative recurrence and rapid, uncontrolled growth.

**Case presentation:**

A 74-year-old man presented to our hospital with complaints of abdominal fullness and pain after eating. On admission, it was observed that the peripheral leukocyte counts and serum G-CSF levels were significantly elevated (23,770/µL and 251 pg/mL, respectively). Computed tomography of the abdomen revealed a pancreatic head tumor involving the superior mesenteric vein. Pathologically, ultrasound-guided fine-needle aspiration confirmed ACP. Subsequently, we performed a subtotal stomach-preserving pancreaticoduodenectomy with portal vein reconstruction and partial transverse colon resection. On postoperative day (POD) 7, the leukocyte count decreased from 21,180/μL to 8490/μL; moreover, computed tomography revealed liver metastasis. Therefore, mFOLFILINOX chemotherapy was initiated on POD 30. However, the tumor exhibited rapid progression, and the patient died on POD 45.

**Conclusions:**

G-CSF-producing ACP is rare, and the prognosis of patients is extremely poor. Basic research is required to develop effective drugs against G-CSF-producing tumors, and large-scale studies using national databases are needed to develop multidisciplinary treatment methods.

## Background

Anaplastic carcinoma of the pancreas (ACP) is a rare undifferentiated variant of pancreatic carcinoma with a poor prognosis and an average overall survival of 12.8 months [1]. Granulocyte colony-stimulating factor (G-CSF)-producing tumors have been reported in various organs since its initial discovery in 1977 in patients with lung cancer, with markedly increased leukocyte counts [2]. Lung cancer is the most frequently reported G-CSF-producing tumor; however, such cases of gastrointestinal and pancreatic cancers are rarely reported. The prognosis of patients with G-CSF-producing pancreatic cancers is very poor [3, 4]. Here, we report a case of G-CSF-producing ACP that displayed early postoperative recurrence, and grew rapidly and uncontrollably.

## Case presentation

**Fig. 1:**
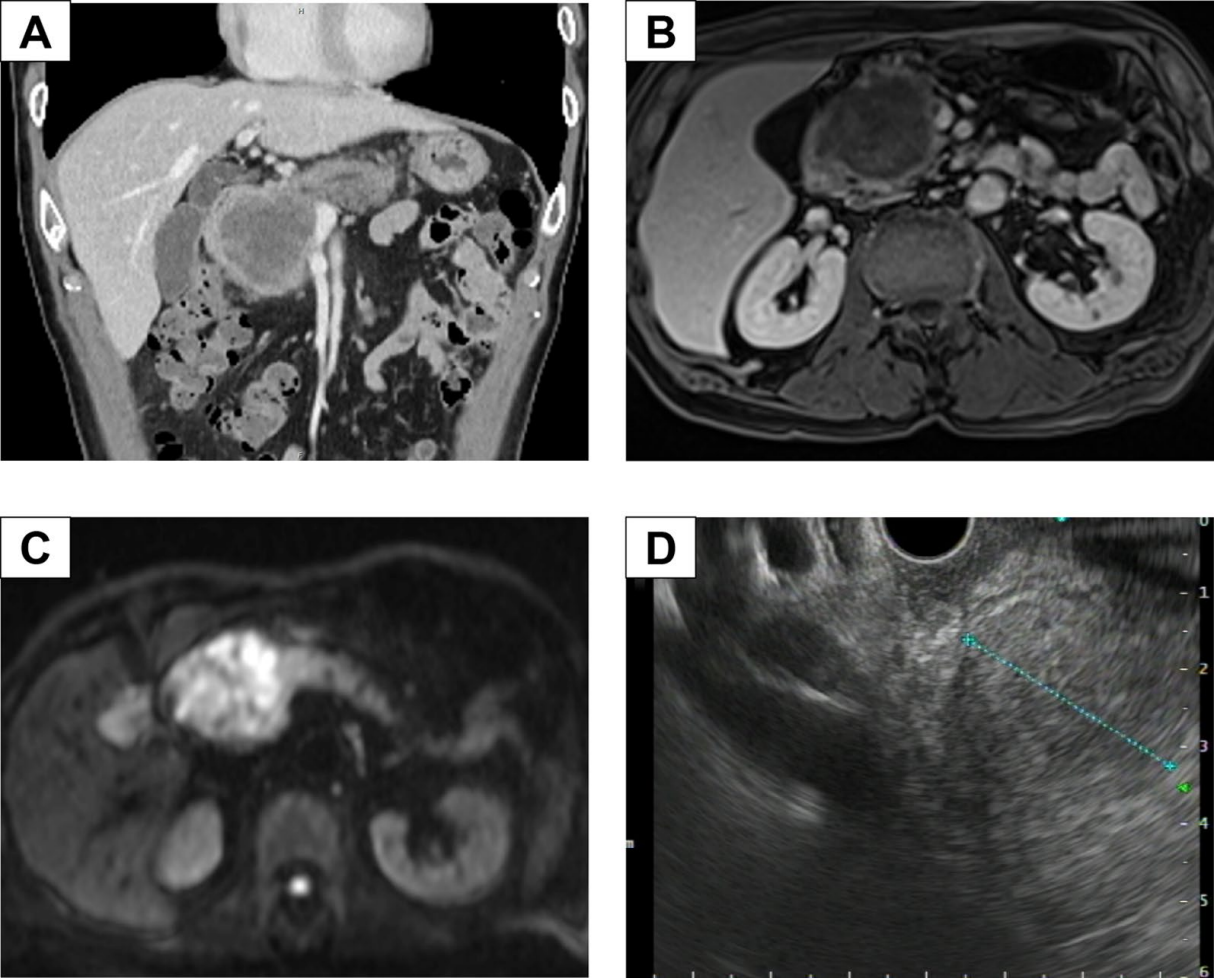
Preoperative images. **A** Abdominal CT in the coronal section revealed a well-demarcated cystic tumor with slight enhancement of the peripheral portion of the pancreatic head. **B** MRI revealed a pancreatic head tumor with SMV invasion. **C** Diffusion-weighted magnetic resonance imaging showing reduced tumor diffusion. **D** Diffusion-weighted images showing a pancreatic head mass with a high signal intensity

**Fig. 2:**
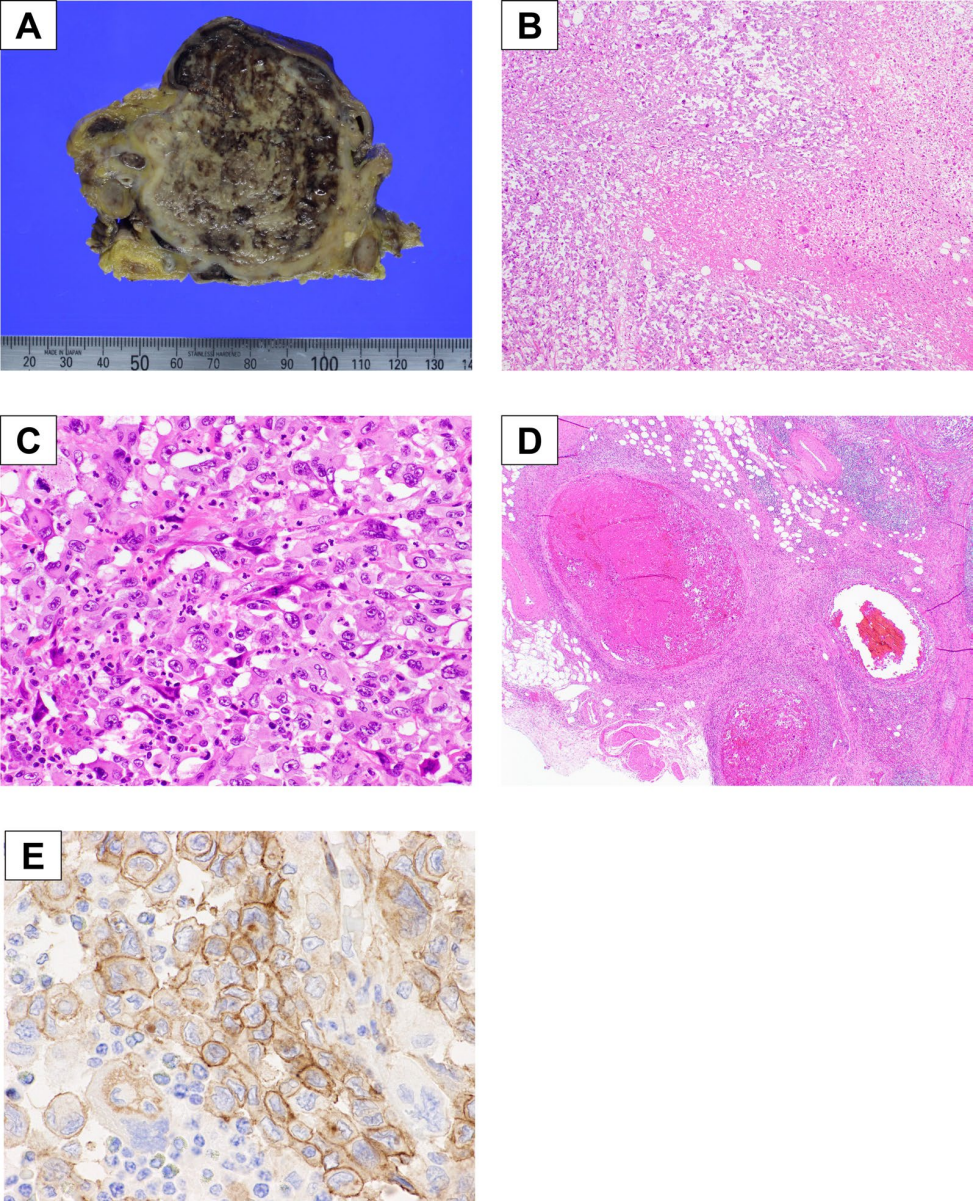
Macroscopic and microscopic findings of the tumor. **A** Macroscopic appearance of the resected specimen. A gray and dark reddish mass measuring 65 mm in diameter with hemorrhage and necrosis was found in the pancreatic head. **B** Histological findings of the tumor showed a diffuse proliferation with necrosis. **C** Tumor cells were poorly cohesive, large, pleomorphic with abundant eosinophilic cytoplasm. The tumor showed a neutrophilic infiltration. **D** Tumor showed a vascular invasion. **E** Immunohistochemical staining for anti-G-CSF antibody in formalin-fixed paraffin-embedded resected specimen. Anaplastic cancer cells were positive for G-CSF

A 74-year-old Japanese man was referred to our hospital with an elevated white blood cell (WBC) count and a pancreatic head tumor measuring 42 mm on computed tomography (CT). He complained of abdominal fullness and pain after eating; however, no jaundice or fever was observed. The patient had a medical history of hyperlipidemia and diabetes mellitus and received the required treatment. After admission, laboratory examination findings were as follows: WBC count, 23,770/μL (90.9% neutrophils, 5.6% lymphocytes, 2.8% monocytes, 0.3% eosinophils, and 0.4% basophils); hemoglobin, 13.6 g/dL; platelet count, 34.7 × 10^4^/mm^3^; C-reactive protein, 7.19 mg/dL). Tumor marker levels were normal: carcinoembryonic antigen, 0.9 ng/mL; carbohydrate antigen 19–9, 17.3 U/mL; duke pancreatic monoclonal antigen type 2, 25 U/mL; and s-pancreas-1 antigen, 5.1 U/mL. The patient exhibited an increased inflammatory response without clinical signs, such as fever; however, antibiotics were administered owing to the possibility of cholangitis. A few days later, a blood test showed a persistently high inflammatory reaction level (WBC: 22,480/μL). G-CSF showed a high value of 251 pg/mL (normal < 39.0 pg/mL). Dynamic enhanced CT, conducted 2 weeks after the previous hospital CT scan, revealed a well-demarcated low-density mass measuring 60 mm in diameter with an enhancement of the peripheral portion of the lesion in the pancreatic head. The tumor involved the superior mesenteric vein (SMV) and gastroduodenal artery (Fig. 1A), but did not invade the nerve of the superior mesenteric artery, and there were no enlarged lymph nodes or distant metastases. Dynamic magnetic resonance imaging (MRI) with Gd-EOB-DPTA enhancement revealed a tumor in the pancreatic head with low intensity on T1-weighted images and slightly high intensity on T2-weighted images. MRI showed clear tumor borders and invasion within the SMV in the portal phase of dynamic enhancement (Fig. 1B). Diffusion-weighted imaging revealed the hyperintensity of the pancreatic head tumor (Fig. 1C). No liver metastases were observed in the hepatobiliary phase. Endoscopic ultrasonography revealed a low-echoic mass with a round shape and capsular-like structures (Fig. 1D). The pathological diagnosis based on ultrasound-guided fine-needle aspiration was anaplastic carcinoma. As there are no reports of effective chemotherapy for anaplastic pancreatic cancer and no clear distant metastasis or non-resectable factors, we decided to perform surgery for pancreatic head cancer without preoperative chemotherapy. Intraoperative findings showed that the tumor had invaded the SMV and transverse mesocolon. Intraoperative Sonazoid contrast-enhanced ultrasonography revealed no obvious liver metastases. Peritoneal dissemination was not observed. We performed a subtotal stomach-preserving pancreaticoduodenectomy (PD) with portal vein reconstruction, partial transverse colon resection, and the modified Child method. The operation time was 406 min, and the blood loss was 650 g. The resected specimen showed a gray and dark reddish brown solid mass with hemorrhage and necrosis measuring 65 mm in diameter in the pancreatic head (Fig. 2A). Histologically, the tumor showed a diffused proliferation with necrosis (Fig. 2B). The tumor comprised poorly cohesive, large, pleomorphic cells with abundant eosinophilic cytoplasm and a neutrophilic infiltration (Fig. 2C). The tumor showed venous invasion (Fig. 2D). Immunohistochemical analysis of the resected specimen revealed G-CSF expression on the plasma membrane of tumor cells (Fig. 2E). Pathological diagnosis was Ph, TS4 (65 mm), nodular type, anaplastic carcinoma, pleomorphic type, int, INFb, Ly1, V2, Pn2, mpd0, pT3, pCH0, pDU1, pS1, pRP0, pPV1 (PVsm), pA0, pPL0, pOO0, pPCM0, pBCM0, pDPM0, R0, pN1a (1/16) M0 pStage IIB, according to the JPS 8th classification. It was classified as T3, N1, M0, Stage IIB according to the UICC 8th TNM staging system. We finally diagnosed the case as G-CSF-producing pleomorphic ACP based on these pathological findings. The WBC level decreased from 21,180/μL on the day before the surgery to 8490/μL on the postoperative day (POD) 7. Dynamic CT on POD 7 showed no obvious intra-abdominal complications, but did reveal liver metastasis in hepatic segment IV, measuring 8 mm in diameter. The patient required rehabilitation to improve the quality of daily life before discharge. The patient progressed without any postoperative complications and was discharged on POD 21.

**Fig. 3:**
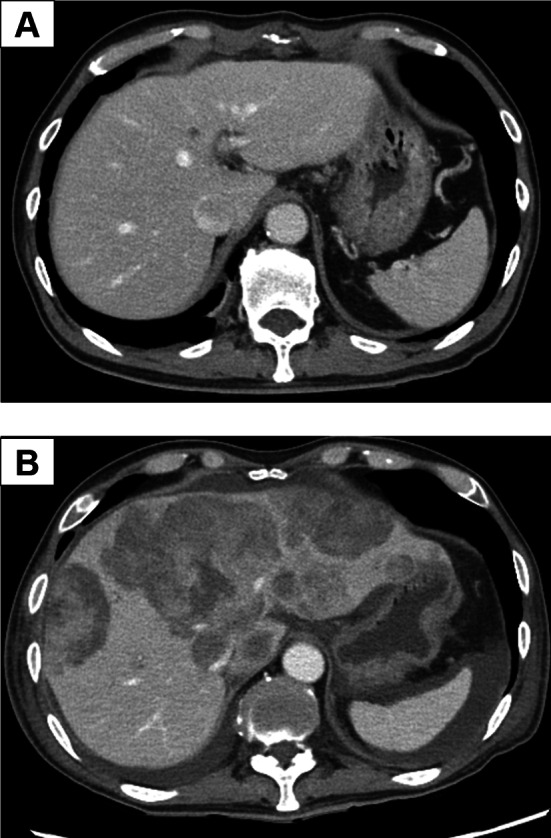
Postoperative enhanced abdominal computed tomography findings. **A** CT showing an 8 mm nodule on the hepatic segment 4 on POD 7. **B** Tumor in liver segment 4 increased to 85 mm on POD 41. Multiple intrahepatic liver metastases were also observed

A blood test on POD 29 showed a high WBC count of 33,010/μL; however, no fever or other inflammatory reactions were observed. Modified FOLFILINOX was initiated on POD 30 as chemotherapy for recurrence. On POD 39, the patient was admitted to the hospital with a loss of appetite and fatigue. On POD 41, the patient had impaired consciousness, and a head CT scan showed multiple cerebral infarcts, leading to a diagnosis of Trousseau’s syndrome. Abdominal CT revealed that the metastatic liver tumor had spread rapidly (Fig. 3A, B). Treatment was set as the best supportive care; however, the patient died on POD 45.

## Discussion

This report describes a rare case of G-CSF-producing ACP. The frequency of ACP is reported to be 0.1–5.7% [3]. The median overall survival of patients with ACP is poor (< 1 year), except for surgical cases [4]. A recent report highlighted an aggressive resection of a huge ACP, including other invaded organs, with an improved prognosis [5]. G-CSF-producing ACP is extremely rare, with only a few case reports, and the prognosis is very poor in all the reports [6–14]. Of the 345 pancreatic cancer cases operated on in our hospital over the past 10 years, 8 (2.3%) were ACP cases, of which only 1 (0.3%), the present case, was G-CSF-producing. The following four characteristics of G-CSF-producing tumors were presented by Asano et al. [2]. (i) extreme leukocytosis, (ii) elevated G-CSF activity, (iii) decreased WBC count after tumor resection, and (iv) detection of G-CSF production in the tumor [2]. They have also been described as the diagnostic criteria in previous reports [11]. In one case from Table 1, G-CSF was not measured, but immunostaining results showed G-CSF production along with other features, and it was diagnosed as a G-CSF-producing tumor [14]. In another case, the WBC count did not improve postoperatively; however, in one case of R1 resection, the postoperative WBC count remained high and displayed early recurrence, and it was thus diagnosed as a G-CSF-producing tumor [8]. Currently, tumors are diagnosed as G-CSF-producing tumors even if all the four diagnostic criteria are not fulfilled. In the present case, all diagnostic criteria were met. There have been reports of G-CSF production in other types of pancreatic cancers, such as poorly differentiated adenocarcinomas [15] and adenosquamous carcinomas [16]. Mucinous cystic neoplasms [17] and solid pseudopapillary tumors [18] that produce G-CSF have also been reported.

**Table 1:** List of resected case of G-CSF producing ACP

Author and year	Age	Gender	Symptoms	WBC	G-CSF (pg/mL)	WBC decrease after surgery	G-CSF detection in tumor	Location	Tumor size (mm)	Histology type	Operation	Postoperative therapy	Survival (days)
Uematsu, 1996 [7]	64	M	Fever	24,300	157	+	+	Tail	70	Anaplastic	DP	–	56
Ando, 2005 [8]	62	F	Fever, general fatigue	15,700	151	−	+	Tail	–	Anaplastic giant cell type	DP, PC	–	38
Gotohda, 2006 [6]	46	M	General fatigue	14,300	155	+	+	Tail	–	Anaplastic	DP, TG	–	120
Murata, 2009 [9]	59	M	Epigastralgia	29,500	110	+	+	Body and tail	–	Anaplastic	DP	GEM + radiation	8 months
Inoue, 2012 [10]	70	F	Epigastralgia	34,500	337	+	+	Tail	90	Anaplastic pleomorphic type	DP, PG, PC	GEM + radiation	8 months
Kitade, 2015 [11]	68	M	Fever, weight loss	17,500	355	+	+	Tail	154	Anaplastic pleomorphic type	DP	S-1 steroid	83
Kudo, 2015 [12]	65	M	Fever	27,900	165	+	+	Tail	70	Anaplastic	DP, PC	S1, GEM	116
Vinzens, 2017 [13]	67	M	Weight loss, upper abdominal pain	25,200	43	+	+	Tail	135	Undifferentiated anaplastic	DP PG, PC	Oxa, IRI, 5-FU	34
Seki, 2018 [14]	65	M	Not described	12,700	–	+	+	Head	55	Anaplastic	PD	–	58
Our case, 2023	74	M	Abdominal fullness, abdominal pain	23,770	251	+	+	Head	65	Anaplastic pleomorphic type	SSPPD, PC	Oxa, IRI, 5-FU	45

*ACP* anaplastic carcinoma of the pancreas, *WBC* white blood cell, *G-CSF* granulocyte-colony stimulating factor, *DP* distal pancreatectomy, *PC* partial colectomy, *TG* total gastrectomy, *PG* partial gastrectomy, *PD* pancreaticoduodenectomy, *SSPPD* subtotal stomach-preserving *PD GEM* gemcitabine, *Oxa* oxaliplatin, *IRI* irinotecan

Reports on other organs also suggest poor prognosis of G-CSF-producing tumors [19, 20]. *KRAS* mutant allele-specific imbalance correlates with the progression to ACP [21], and G-CSF expression correlates with *KRAS* mutations in pancreatic tumors [22]. G-CSF enhances pancreatic cancer cell proliferation via autocrine signaling by increasing inflammatory cytokines, adversely affecting cancer progression [23]. In this case, two preoperative and two postoperative CT scans were performed, which confirmed tumor growth within a short period. Pre-operatively, the tumor doubling time was 8 days, whereas the postoperative liver metastatic lesion showed faster tumor growth, with a doubling time of 3 days. The inflammatory response to surgical invasion possibly stimulates tumor growth. Epithelial–mesenchymal transition (EMT) is associated with poor prognosis in ACP [4]. Decreased E-cadherin and increased N-cadherin expression have previously been associated with EMT, which mediates cancer progression [24]. Immunohistochemical staining showed that E-cadherin expression was absent in seven out of eight cases of anaplastic carcinoma [25].

**Fig. 4:**
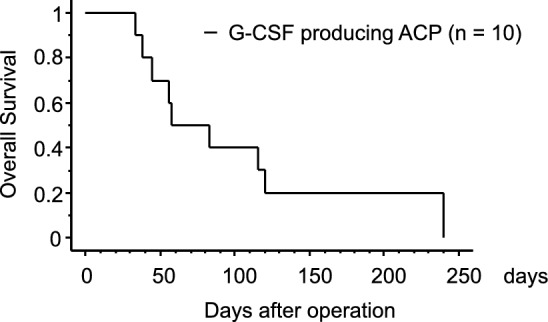
Kaplan–Meier curve for G-CSF producing ACP cases. Survival curve for previous resected cases of G-CSF producing ACP are shown. Median survival time was only 58 days

A search of the PubMed database using the keywords ‘ACP or undifferentiated carcinoma’ and ‘G-CSF’ was performed. Nine cases of surgical resection of this rare high-grade tumor have been reported, with the present case being the tenth; however, there may be additional cases. The prognosis has been consistently poor (Table 1) [6–14]. The possibility that only cases with extremely poor outcomes have been reported, cannot be denied. Among these, there were eight male patients, with many cases involving large tumors, averaging 91 mm (55–154 mm) in diameter. In two cases, G-CSF levels were low or not measured, but the diagnosis was based on the criteria for G-CSF-producing tumors. PD was performed for lesions in the pancreatic head only in this case and in one other case. Complicated resection of other organs was performed in 6 of the 10 cases, probably because of the large tumor size and invasion. Patients with pleomorphic pathologies tend to have high G-CSF levels. A patient with reduced accumulation of fluorodeoxyglucose with TS-1 treatment survived for only 88 days [26]. Inoue et al. reported that radiotherapy temporarily reduced the size of recurrent lesions after surgery, suggesting that radiotherapy might be effective [10]. However, while there are a few case reports suggesting potential effectiveness, no clear treatment strategy has been established. All patients died within 8 months, and most cases recurred early in the immediate postoperative period. The median survival time for ACP patients with G-CSF production was only 58 days by survival curves using the Kaplan–Meier method (Fig. 4). The postoperative prognosis for G-CSF-producing ACP was very poor. This rare and highly malignant tumor has been treated by surgical resection only 10 times, with consistently poor outcomes. We expect that a multidisciplinary treatment approach, including surgery, chemotherapy, and radiotherapy, will improve prognosis. Additionally, further basic research can contribute to the development of effective treatment methods for G-CSF-producing tumors. There has been no large-scale analysis of the prognosis for G-CSF-producing ACP using national databases, making it necessary to further investigate this rare cancer to develop potential treatment strategies.

## Conclusions

A very rare case of G-CSF-producing ACP was reported. Extended surgical resection was performed; however, early recurrence and rapid proliferation led to unfortunate outcomes. No case has been reported of a prolonged prognosis, and the disease urgently requires the development of new treatment strategies.

## Data Availability

Data sharing was not applicable to this article as no datasets were generated or analyzed during the current study.

## Abbreviations

ACP: Anaplastic carcinoma of the pancreas
G-CSF: Granulocyte-colony stimulating factor
WBC: White blood cell
CT: Computed tomography
SMV: Superior mesenteric vein
MRI: Magnetic resonance imaging
PD: Pancreaticoduodenectomy
POD: Postoperative day
EMT: Epithelial–mesenchymal transition
